# The impact of hyperoxia on brain activity: A resting-state and task-evoked electroencephalography (EEG) study

**DOI:** 10.1371/journal.pone.0176610

**Published:** 2017-05-02

**Authors:** Min Sheng, Peiying Liu, Deng Mao, Yulin Ge, Hanzhang Lu

**Affiliations:** 1 Advanced Imaging Research Center, The University of Texas Southwestern Medical Center, Dallas, Texas, United States of America; 2 Department of Radiology, Johns Hopkins University School of Medicine, Baltimore, Maryland, United States of America; 3 Department of Radiology, New York University Langone Medical Center, New York, New York, United States of America; Penn State University, UNITED STATES

## Abstract

A better understanding of the effect of oxygen on brain electrophysiological activity may provide a more mechanistic insight into clinical studies that use oxygen treatment in pathological conditions, as well as in studies that use oxygen to calibrate functional magnetic resonance imaging (fMRI) signals. This study applied electroencephalography (EEG) in healthy subjects and investigated how high a concentration of oxygen in inhaled air (i.e., normobaric hyperoxia) alters brain activity under resting-state and task-evoked conditions. Study 1 investigated its impact on resting EEG and revealed that hyperoxia suppressed α (8-13Hz) and β (14-35Hz) band power (by 15.6±2.3% and 14.1±3.1%, respectively), but did not change the δ (1-3Hz), θ (4-7Hz), and γ (36-75Hz) bands. Sham control experiments did not result in such changes. Study 2 reproduced these findings, and, furthermore, examined the effect of hyperoxia on visual stimulation event-related potentials (ERP). It was found that the main peaks of visual ERP, specifically N1 and P2, were both delayed during hyperoxia compared to normoxia (P = 0.04 and 0.02, respectively). In contrast, the amplitude of the peaks did not show a change. Our results suggest that hyperoxia has a pronounced effect on brain neural activity, for both resting-state and task-evoked potentials.

## Introduction

Oxygen is critical to human life. Due to the relative convenience and low-cost to modulate O_2_ levels, manipulation of its concentration in the body via control of inspired air has generated opportunities for clinical evaluation and interventions. Oxygen therapy has been widely used in wound-healing from radiation injury and diabetes [1,2], and every day, thousands of people receive oxygen treatment in major medical centers and private practice facilities. However, despite the increasing use of oxygen as a treatment, the effect of O_2_ gas modulation on the brain is poorly understood. Furthermore, certain applications of oxygen therapy specifically target brain diseases, such as cerebral ischemia [3–6], traumatic brain injury [7,8], and cerebral palsy [9]. While the premise of many of these efforts is based on the metabolic need for hyperoxic gas, i.e., delivering more oxygen to the brain tissue, there could be other effects of hyperoxia on the brain. The goal of the present study was, therefore, to characterize the effect of hyperoxia on neural activity in healthy subjects, in whom oxygen delivery is sufficient under normoxic conditions.

In addition to therapeutic applications, hyperoxia has also been used to calibrate the Blood-Oxygen-Level Dependent (BOLD) signal in functional magnetic resonance imaging (fMRI) to better estimate baseline and evoked neural metabolism in the human brain [10–12]. However, in this approach, an important assumption is that hyperoxia itself does not alter neural activity. Therefore, a better understanding of the effect of hyperoxia on neural activity may improve fMRI technologies in terms of providing more accurate brain mapping.

Changes in neural activity induced by hyperoxia cannot be examined by indirect approaches, such as fMRI, Positron Emission Tomography (PET), or functional optical imaging, because those methods cannot differentiate the neural effects from the vascular effects of hyperoxia. Consequently, several reports have focused on measures that are more directly related to neural activity [13,14]. Xu et al. [13] measured the cerebral metabolic rate of oxygen (CMRO_2_) and observed a decrease in this rate under hyperoxia. Prior studies have also measured neural activity during hyperoxia, with mixed results. Kaskinoro et al. measured EEG under hyperoxia and reported an absence of signal change compared to normoxia [15]. In contrast, Croal et al. used magnetoencephalography (MEG) and observed a moderate reduction (3–5%) in occipital lobe signal power [16]. However, none of the prior studies differentiated the effects of hyperoxia during resting-state versus task-evoked activities.

In the present work, we used EEG to measure resting-state and task-evoked neural activity in a group of healthy subjects and examined EEG signal differences between hyperoxia and room-air conditions.

## Materials and methods

### Participants

The Institutional Review Board of the University of Texas Southwestern Medical Center approved the study protocol. The studies were conducted between January 28, 2013 and December 20, 2013. Thirty-nine healthy subjects, 19–46 years of age (30.0±7.3 years) were enrolled. Twenty-six subjects were enrolled in the resting state EEG study, 13 of whom (30.0±6.7 years, seven male, six female) were assigned to the O_2_-breathing group and the other 13 (30.4±8.2 years, seven male, six female) were assigned to the sham control group. Thirteen subjects (29.7±7.7 years, six male, seven female) were enrolled in the task-evoked EEG study. The order of the room-air and O_2_-breathing conditions were counterbalanced across participants in the task-evoked study, thus a sham study was not performed. The participants were recruited from the University campus through flyers. None of the participants reported a history of neurological or psychiatric disorders, or pulmonary conditions, such as asthma. Participants were asked not to consume any caffeine-containing beverages for one hour before the experiment. All participants gave informed, written consent before participating in the study.

We conducted a power calculation based on the typical sensitivity of our EEG system in which a one-minute EEG signal has a coefficient-of-variation (CoV) of approximately 20%. Based on our previous study of brain metabolic changes during hyperoxia, we assume that the EEG signal will change by 15% [13]. We estimated that, with a sample size of N = 13, we will have 0.95 power to detect an EEG signal change in our study when comparing O_2_-breathing to room-air condition.

### General experimental procedures

**Fig 1:**
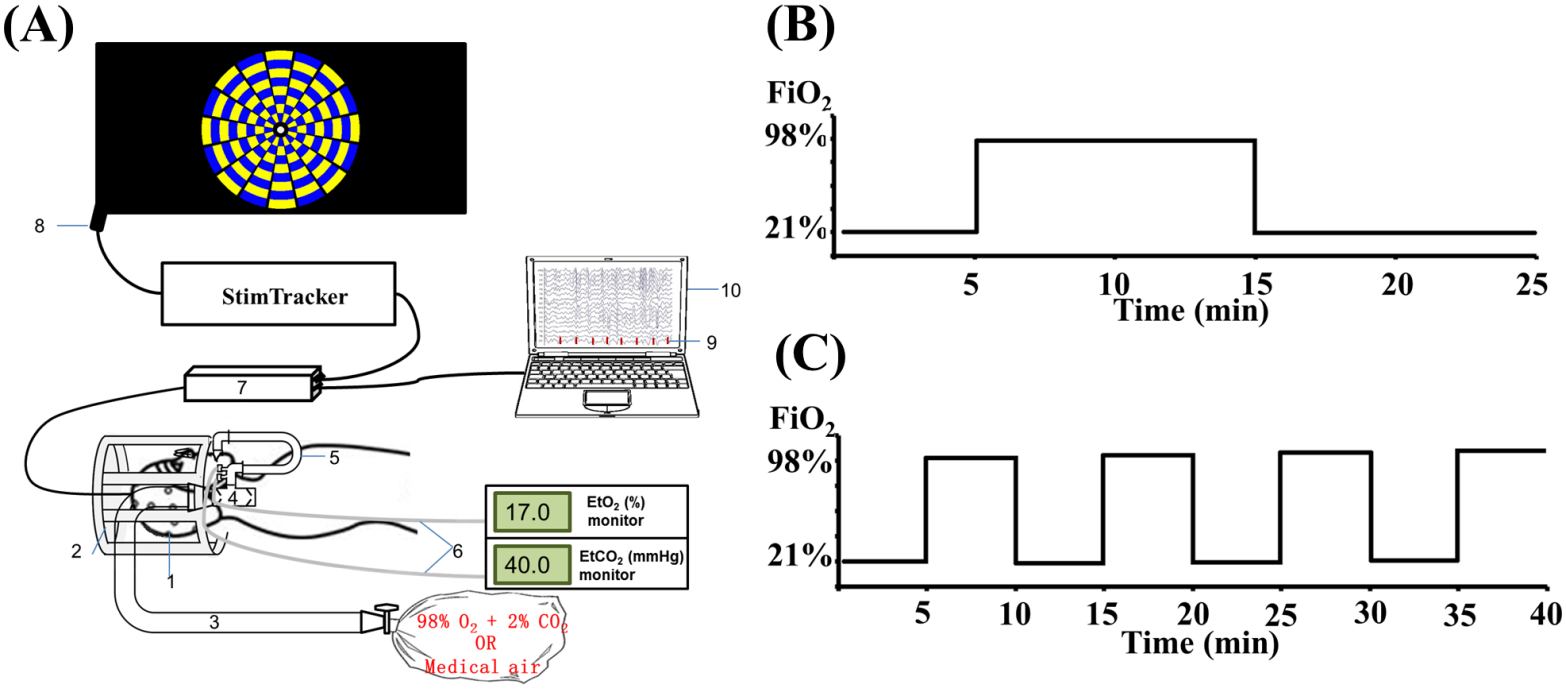
Study procedure and paradigm. (a) EEG experimental setup. Components of the setup are labeled in the diagram and indicate the following: 1. EEG cap; 2. Mock coil to support the breathing valve; 3. Gas delivery tube; 4. Two-way non-rebreathing valve; 5. U-shaped tube; 6. Gas sampling tubes (one for CO_2_ and the other for O_2_); 7. EEG amplifier; 8. Stimulus tracker for accurate recording of stimulus onset time; 9. Stimulus markers; and 10. EEG recording laptop. (b) Timing paradigm of the resting EEG study. (c) Timing paradigm of the task-evoked EEG study.

Hyperoxia was achieved with a breathing apparatus used extensively in our laboratory (http://www.mri.jhmi.edu/hlulab/index_files/Page363.html) [17–20]. The hyperoxic gas consisted of 98% oxygen and 2% CO_2_ contained in a Douglas bag (Fig 1A). The reason the hyperoxic gas contained a small amount of CO_2_ is that hyperoxia tends to cause hyperventilation, which could result in a decrease in EtCO_2_, if not accounted for. Thus, by adding a small amount of CO_2_ in the inspired gas, the EtCO_2_ value can be maintained relatively constant if subjects hyperventilate. The proper amount of CO_2_ that can offset the hyperventilation effect but not cause an increase in EtCO_2_ was previously calibrated. EtCO_2_ results shown in Table 1 confirmed that there were minimal changes in EtCO_2_ during the oxygen challenge.

**Table 1:** Summary of results of the resting-state EEG study (Mean ± SEM).

	O_2_-breathing group			Sham control group		
Age (yrs)	30.0 ± 1.8			30.4 ± 2.3		
Gender	7 Males, 6 Females			7 Males, 6 Females		
	Normoxia	Hyperoxia	P value	Normoxia	Medical air	P value
EtO_2_ (mmHg)	138.3 ± 0.8	700.7 ± 3.0	<0.001	136.0 ± 0.8	136.8 ± 0.8	0.08
EtCO_2_ (mmHg)	37.4 ± 4.4	37.5 ± 2.4	0.85	39.3 ± 1.1	39.5 ± 1.1	0.67
Comfort level	1.83 ± 0.2	1.93 ± 0.2	0.43	1.92 ± 0.2	1.88 ± 0.2	0.68
Response time (ms)	542.1 ± 17.4	557.7 ± 23.3	0.17	560.3 ± 15.1	564.6 ± 15.7	0.65

The P values represent results of paired Student t tests between normoxia and the gas mixture conditions.

The experiment was conducted with the participant in a supine position in a quiet mock-MRI room, in which visual stimulation and breathing comfort-level rating questions could be delivered via a back-projection display, and subject responses could be recorded through a button box. The participant breathed through a mouthpiece that was connected to a two-way valve (Hans Rudolph, 2600 Series, Shawnee, KS). A nose clip was used to block nasal breathing to ensure the control of inhaled air. The participant was instructed to avoid biting on the mouthpiece, which could result in artifacts in the EEG signal due to an electrical signal from the facial muscles. EtCO_2_ and EtO_2_ were recorded continuously during the entire session using a capnograph device (Capnogard, Model 1265, Novametrix Medical Systems, CT) and an O_2_ sensor (Biopac, Module O_2_ 100C, Biopac Systems, CA). One researcher remained inside the recording room throughout the experiment to monitor the physiology of the participant and to switch the valve to change the inspired gas. The participant was not aware of the timing of the gas switches, as the gas valve was outside the bore.

During the experiment, the participant was asked (via the back-projection display) to rate their comfort level of breathing every one minute, on a scale of 1 to 5, with 1 indicating very comfortable and 5 indicating very uncomfortable.

### EEG recording

A 32-channel EEG system (Brain Products, Gilching, Germany) was used to perform the EEG experiments. After the EEG cap was placed on the head of the participant, high-chloride abrasive electrolyte gel (Brain Products, Gilching, Germany) was applied on each electrode such that the impedance values of all electrodes, except for the electrocardiogram (ECG) electrode, were below 10kΩ. The participant was then instructed to lie down on the table, and a mouthpiece and nose clip were placed. The EEG recording was then initialized and continued through the entire session across all respiratory states.

### Resting-state study (Study 1)

In the resting state EEG study, each participant underwent a 25-minute session, as shown in Fig 1B, in which the participant breathed room-air during the first five minutes, followed by ten minutes of breathing a gas mixture contained in a Douglas bag, and then returned to room-air breathing during the final ten minutes. For participants in the O_2_-breathing group, the Douglas bag contained a hyperoxic gas mixture of 98% O_2_ and 2% CO_2_. For those in the sham-control group, the bag contained 21% O_2_ and 79% N_2_, equivalent to the composition of room-air. The participants of both groups were given the same instructions (both were told they would inhale oxygen).

A sustained-attention measure was collected throughout the session to verify that any EEG alteration was not due to a change in attention. The sustained-attention measure was relatively low-demand and comprised 26 alphabetical letters appearing in the center of the screen sequentially, in a randomized order. The participant was instructed to press a button in their right hand as quickly as possible when they saw a target letter, such as ‘m.’ The duration each letter appeared on the screen was 500ms. The size of the font was relatively small and was not expected to induce any visual evoked potentials. Response time, which is the time gap between the display onset of the letter and the button press, was recorded and sorted by respiratory conditions. Thus, it should be pointed out that the resting-state study was not performed under completely “resting” conditions. However, at least the mental state of the participants was under controlled conditions.

The EEG data were recorded at a sampling rate of 5000 Hz. Pre-processing of the EEG data was performed using a vendor-provided software Analyzer v2.0 (Brain Products, Gilching, Germany), which included the following steps. 1) Band-pass filtering of 0.3 to 75Hz was applied with additional 60Hz notch filtering. 2) The signal of each electrode was re-referenced to the average potential of all electrodes. 3) Independent component analysis was applied to remove eye-blinking artifacts, and (4) cardioballistic correction was used to eliminate pulse artifacts. Visual inspection was conducted to manually exclude epochs with biting signal artifacts.

Post-processing of the EEG data was performed using Matlab R2008b (Mathworks, Natick, MA). Specifically, after linear detrending, the entire EEG recording was divided into one-second segments and Fast Fourier transform (FFT) was applied to each segment and for each electrode. The power spectrum from 1 to 75Hz was computed in units of μV^2^ at each Hz. The power spectra were then divided into standard frequency bands: 1 to 3Hz for the δ band; 4 to 7Hz for the θ band; 8 to 13Hz for the α band; 14 to 35Hz for the β band; and 36-75Hz for the γ band [21]. Next, the EEG power was averaged across the 31 channels. Finally, EEG power under each physiological state was quantified. The power under room-air was calculated as the average of the first room-air period (five minutes) and the steady-state portion of the second room-air period (the last seven minutes of the second room-air period, excluding the transition period). The first and second room-air periods were averaged to account for potential drift of electrode impedance with time. The power under gas-mixture breathing used the steady-state portion of the gas challenge period (the last seven minutes of the gas-challenge, excluding the transition period). The EEG powers were compared across physiological states using paired t tests. The O_2_-breathing and sham control data were also analyzed together in a two-way replication ANOVA analysis to determine whether the observed effects were specific to the O_2_ group.

### Task-evoked study (Study 2)

In the task-evoked EEG study, the subject breathed four periods of room air (five-minute per period) and four periods of hyperoxic gas (for five minutes) in an interleaved fashion (Fig 1C). The order of room-air and hyperoxia periods was counterbalanced across subjects, thus no sham arm was used in the task-evoked study. During each block, the first two minutes contained no task (only a fixation crosshair was shown on the screen) as it represents a transition period between physiological states. During the last three minutes of the block, 96 visual stimulation trials were presented, in each of which a blue-yellow checkerboard (E-Prime software, Psychology Software Tools) was displayed for 500 ms, followed by a crosshair with a randomized duration between 900 and 1400 ms. To maintain the attention of the participant, the center of the checkerboard (Fig 1A) contained different geometric shapes and the participant was instructed to press the right-hand button if it was a circle and the left-hand button if it was any other shape. The reason we used different shapes rather than different letters, as in the resting-state study, is that the large number of letters would result in a long interval between matches, which is not ideal for maintaining a subject’s attention. The total number of button presses between the two hands was balanced.

The digital output of the video display often causes an uncertainty in the onset time of the stimulus, which is related to the refreshing frequency of the display. To obtain a better accuracy with regard to stimulus onset, we applied a photo sensor (Stim tracker, Cedrus Corporation, San Pedro, CA) on the corner of the screen, the timing of which was recorded as an event marker on the EEG system. This improved the accuracy of the EEG timing to 0.2 ms.

Pre-processing of the task-evoked EEG data followed steps similar to those used for the resting-state EEG. Post-processing was performed using two Matlab-based toolboxes, EEGLab (UC San Diego, USA) and ERPLab (UC Davis, USA). For quantification of amplitude and temporal features of the event-related potentials (ERP), the continuous EEG data were segmented into trials based on the stimulus onset. The data for each trial consisted of EEG recordings from 100 ms before stimulus onset to 500 ms after stimulus onset. Trials from the entire session were sorted into room-air and hyperoxic conditions. ERP waveforms under the same respiratory conditions were averaged over trials (approximately 384) to yield a mean waveform. Amplitude and latency of the two most prominent peaks on the ERP waveform, N1 and P2, were quantified using ERPLab. For amplitude, the maximum signal intensity was determined. For latency, the time at which the signal reached 50% peak amplitude was determined [22,23].

The power spectrum of the ERP signal was also examined. Data segments corresponding to the first 500 ms after the stimulus onset were subject to the FFT analysis and signal powers in the δ, θ, α, β, and γ frequency bands were computed. For comparison, a similar analysis was carried out for the signal at 500ms preceding the stimulus onset. Paired t tests were used to compare the results between respiratory conditions.

## Results

The effects of hyperoxia on resting-state and task-evoked EEG were examined in separate groups of young, healthy participants. There were no differences in age across the groups (ANOVA, P = 0.8). All participants were able to complete the study and none reported any discomfort during the experiments.

### Resting-state EEG study (Study 1)

Twenty-six participants were randomly assigned to an O_2_-breathing group (N = 13) or a sham control group (N = 13). End-tidal O_2_ (EtO_2_) and end-tidal CO2 (EtCO_2_) levels of the participants are summarized in Table 1. As can be seen, breathing of hyperoxic gas, but not medical air (21% O_2_ and 79% N_2_), increased EtO_2_. EtCO_2_ was not altered during the hyperoxic condition because of the small amount of CO_2_ added to the hyperoxic gas, which offset the effect of hyperventilation. This ensured that any neural activity alteration that we observed could be attributed to O_2_ changes rather than to a CO_2_ effect. The comfort level of breathing recorded during the session also showed no differences across physiological states (Table 1), suggesting that any neural changes observed during the experiment could not be attributed to an increased attention to breathing.

**Fig 2:**
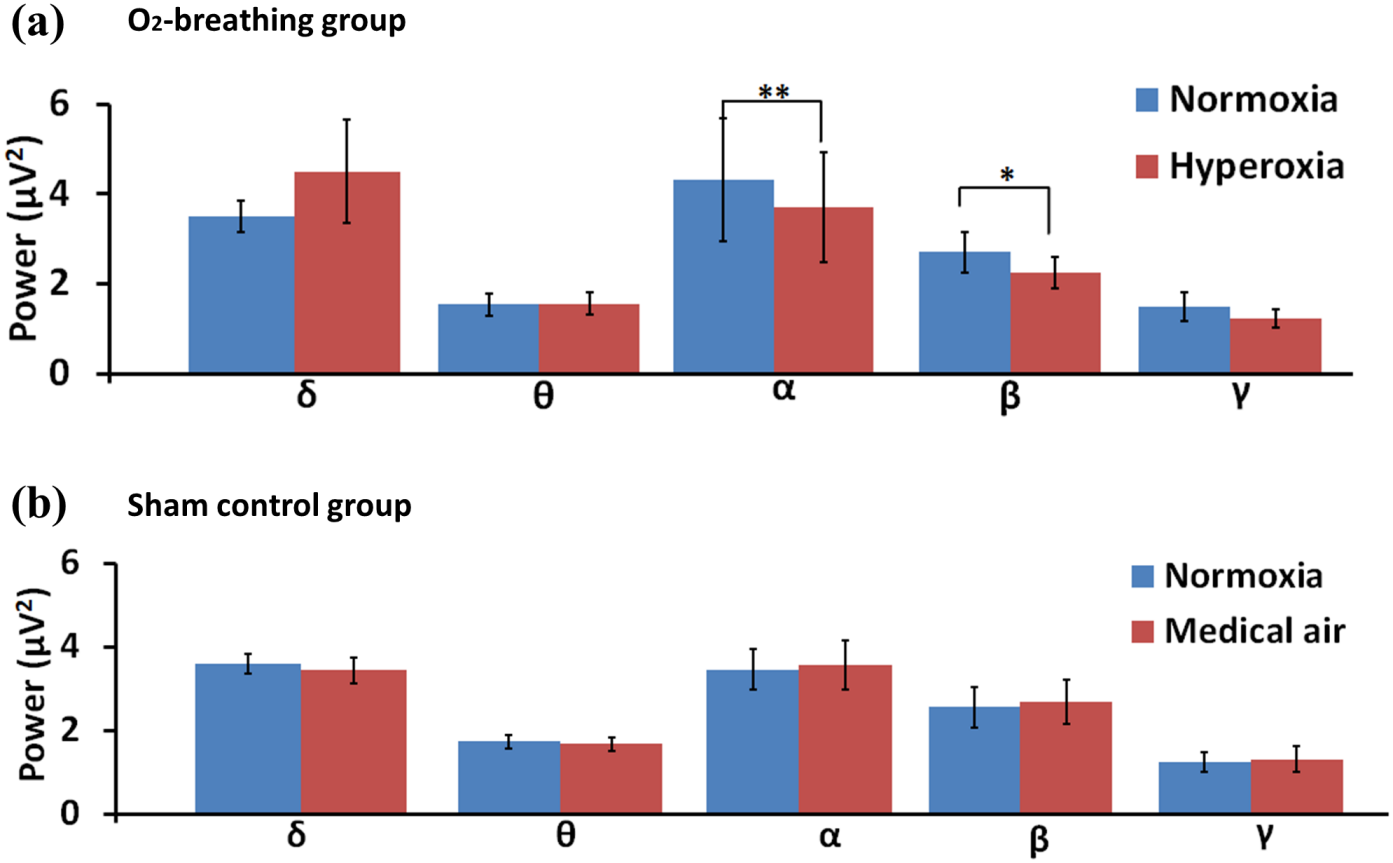
Bar plots of EEG spectral power in (a) O_2_-breathing (N = 13) and (b) sham control (N = 13) experiments. NO = normoxia, HO = hyperoxia, MA = medical air. One asterisk = P<0.05. Two asterisks = P<0.01. Error bar = standard error across participants.

Fig 2 displays resting-state EEG signal power by frequency band for both the O_2_-breathing and the sham control groups (see S1 File for individual subject data). Paired comparison tests between the breathing states revealed that, in the O_2_-breathing group (Fig 2A), the signal power was significantly decreased in the α band (8-13Hz) by 15.6±2.3% (P = 0.003) and in the β band (14-35Hz) by 14.1±3.1% (P = 0.005) (mean ± SEM) when breathing oxygen. The δ (1-3Hz), θ (4-7Hz), and γ (36-75Hz) bands did not show a significant difference (P = 0.3, P = 0.5, and P = 0.3, respectively). The δ band plot (Fig 2A) showed a slight increase in hyperoxia although this did not reach statistical significance. Upon further investigation of the data, it was noted that this increase was due to a single subject who showed a large increase in δ band power. When this data point was omitted from the averaging, the plot showed no change in δ power (see S1 Fig). In the sham control group (Fig 2B), none of the bands showed a significant change in signal power.

**Fig 3:**
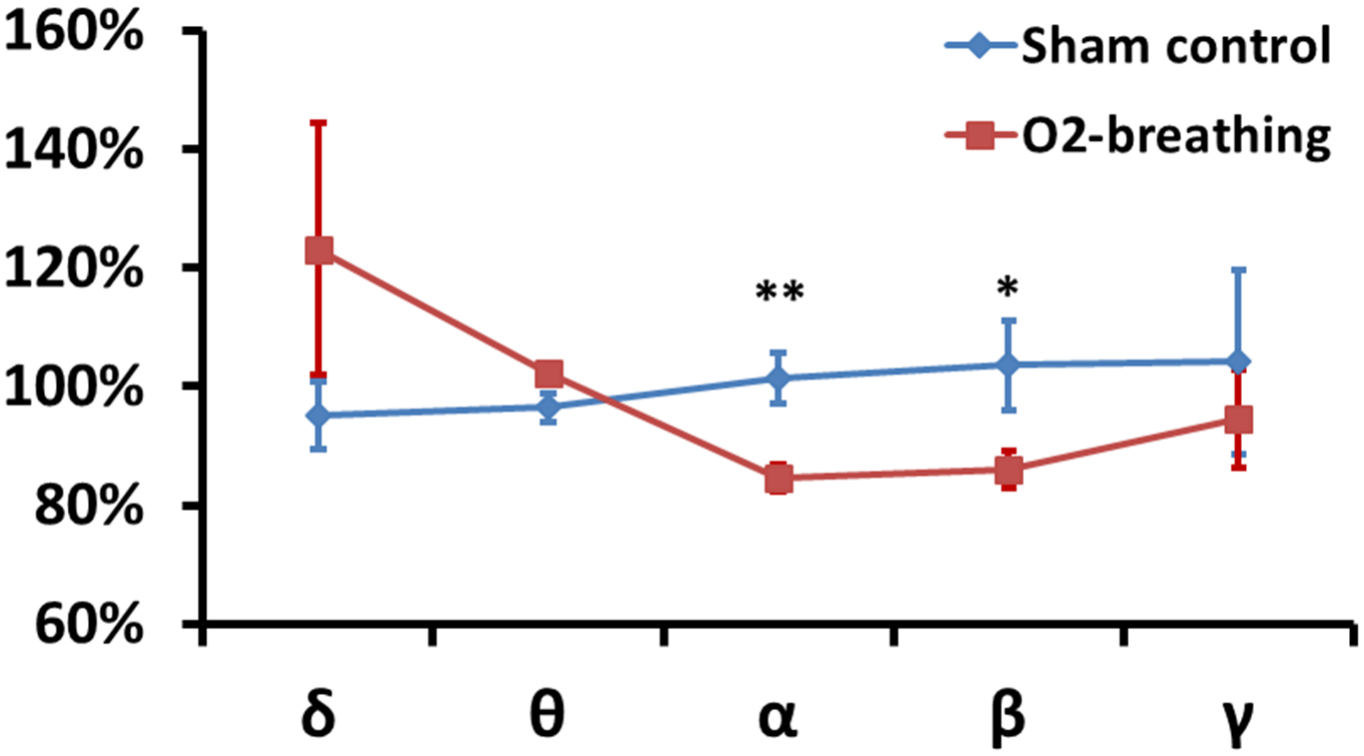
Ratio of EEG signal power for special gas mixture compared to room-air breathing. For the O_2_-breathing experiment, the special gas mixture used hyperoxic gas (98% O_2_ + 2% CO_2_). For the sham control experiments, the special gas mixture used medical air (21% O_2_ + 79% N_2_). One asterisk = P<0.05 for the comparison between red and blue symbols. Two asterisks = P<0.01 for the comparison between red and blue symbols. Error bar = standard error.

Next, the ratio of EEG power between the gas mixture and the room-air periods was computed for both the O_2_-breathing group and the sham control group (Fig 3), which factored out intersubject variations in EEG power. The ratio values were then compared using a two-way replication ANOVA analysis, in which one factor was the frequency bands and the other factor was the study group (O_2_-breathing versus sham control). The group-by-band interaction effect was significant (F = 3.2, P = 0.03), suggesting that the alteration in neural activity was specific to the O_2_-breathing task and was frequency-dependent. Post-hoc t-tests showed that the EEG power ratio in the α (P = 0.002) and β (P = 0.04) bands was significantly different between the O_2_-breathing and the sham control groups (Fig 3).

**Fig 4:**
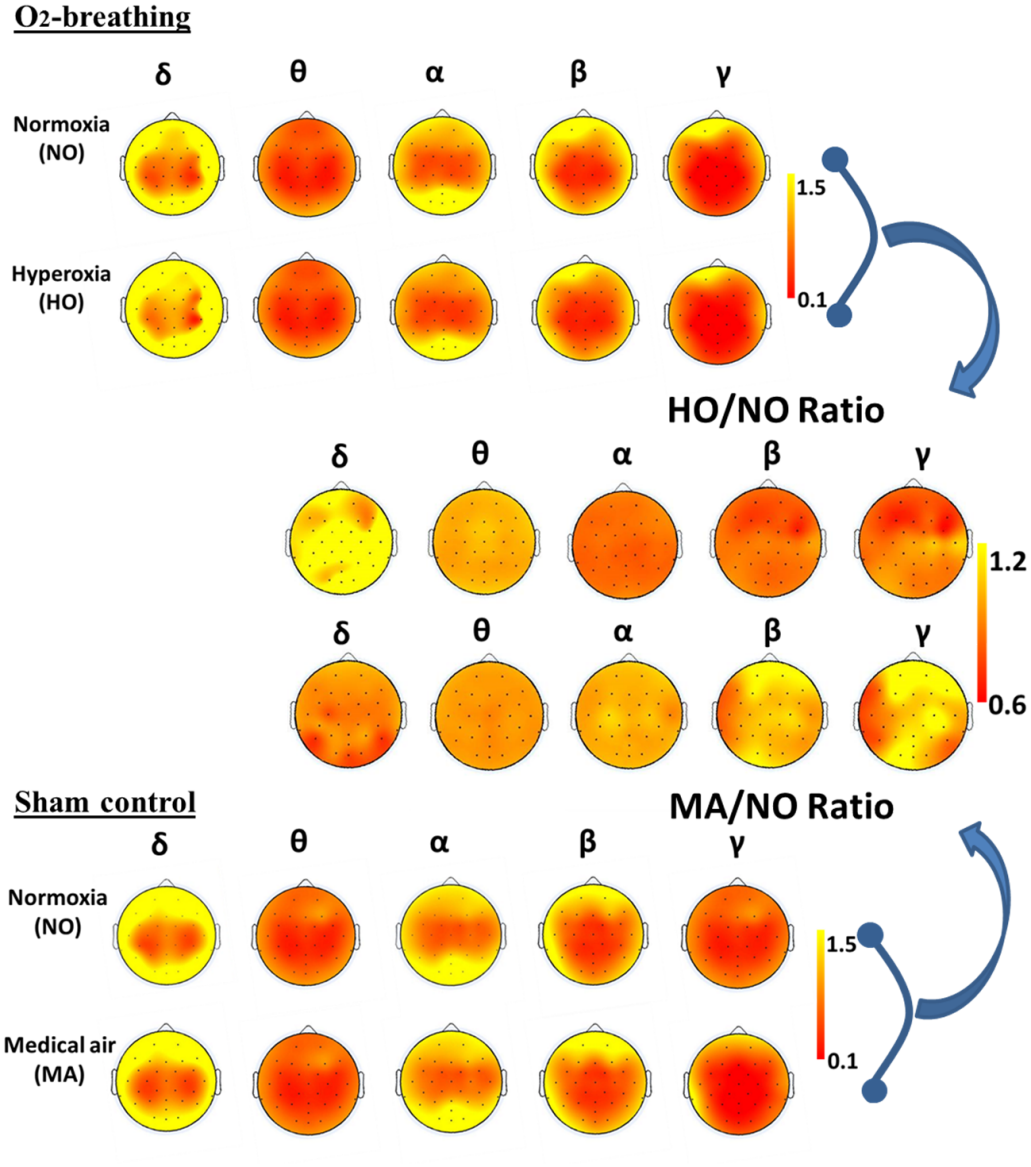
Topographic maps of EEG signals under normoxia and gas-challenge conditions. In the O_2_-breathing experiment, the gas-challenge condition used hyperoxic gas. In the sham control experiment, the gas challenge condition used medical air. The EEG signal power was calculated for each frequency band and the maps shown are averages of all participants. In the topographic maps, the triangle on the top represents the subject’s nose, while two rectangles on each sides represent the ears. Each black dot indicates one electrode. The images on the right-hand side show the ratio between two topographic maps.

Fig 4 shows the group-averaged topographic maps of EEG power, as well as the ratio (gas mixture divided by room-air) of power. It can be seen that the hyperoxic effect on EEG power was widespread across the brain. The higher frequency power was suppressed by O_2_-breathing and the lower frequency components appear to have been enhanced. In contrast, no obvious change was observed in the sham control case.

The level of attention was maintained throughout the experimental session, as there was no difference in response time (P = 0.17) for the attention measure.

### Task-evoked EEG study (Study 2)

**Fig 5:**
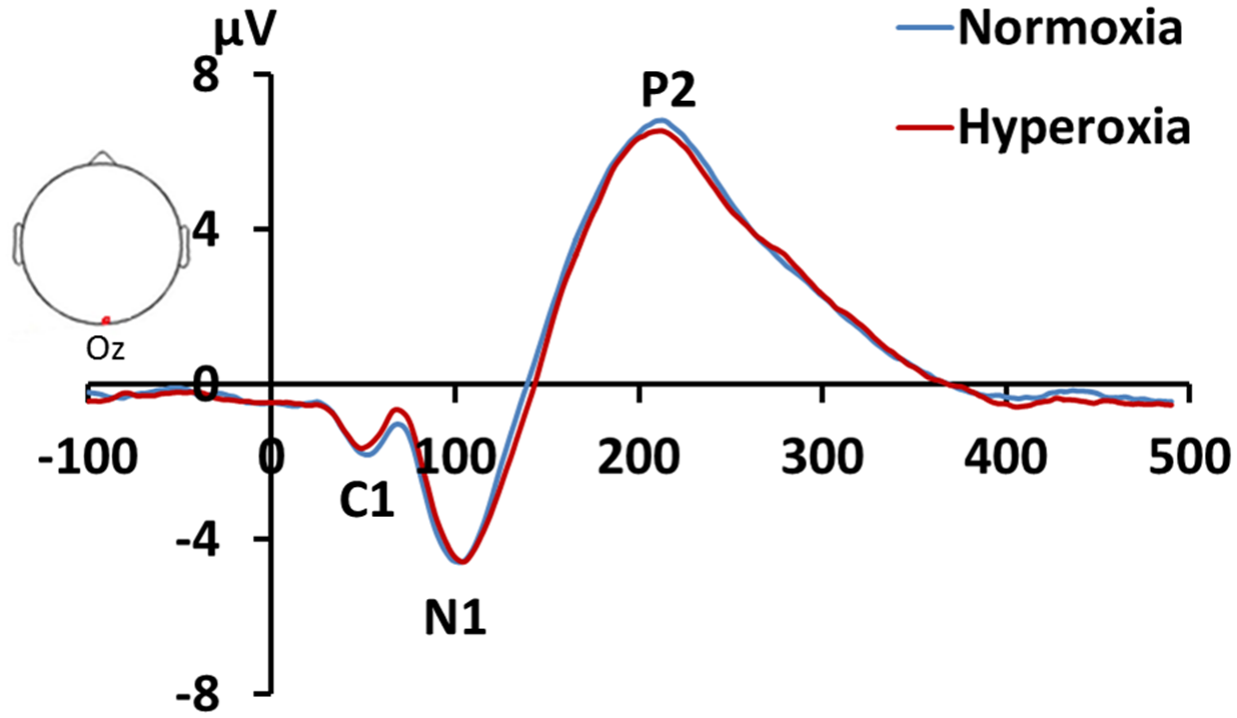
Averaged ERP waveforms (N = 13) in response to a visual stimulus under room-air and hyperoxic conditions. The ERP of the Oz channel is shown. N1 and P2 peaks of the ERP are clearly visible.

Fig 5 shows the ERP waveforms in response to visual stimulation in the EEG electrode that corresponds to the visual cortex, the Oz channel. Several ERP peaks, most prominently N1 and P2, can be clearly identified in every participant. Results of the quantitative analysis are shown in Table 2. There was not a significant difference between room air and hyperoxic air in the ERP response amplitude. However, a difference in response latency was observed (Table 2). Specifically, the ERP response during hyperoxia appears to be slower than that during normoxia. This difference was noted in both the N1 (paired Student t test, P = 0.04) and P2 (P = 0.02) peaks.

**Table 2:** Summary of results of the task-evoked EEG study (mean ± SEM).

	Normoxia	Hyperoxia	P value
Age (yrs)	29.6 ± 2.1		-
Gender	6 Males, 7 Females		-
EtO_2_ (mmHg)	127.7 ± 3.0	688.6 ± 10.6	<0.001
EtCO_2_ (mmHg)	40.0 ± 1.2	39.9 ± 1.0	0.83
Comfort level	2.2 ± 0.2	2.2 ± 0.2	0.53
Response time (ms)	602.7 ± 29.0	594.5 ± 30.1	0.17
N1 peak amplitude (μV)	-6.3 ± 0.9	-6.3 ± 0.9	0.91
P2 peak amplitude (μV)	7.4 ± 0.9	7.3 ± 0.8	0.73
N1 50% peak latency (ms)	88.5 ± 4.5	89.6 ± 4.6	0.04
P2 50% peak latency (ms)	166.8 ± 6.7	168.8 ± 6.6	0.02

The P values represent the results of paired Student t tests between the normoxia and the gas mixture groups.

We further examined the power spectrum of the ERP data. It was found that α and β signal power was significantly decreased by 7.7% ± 3.5% (P = 0.04) and 10.0% ± 2.5% (P = 0.003), respectively, in the hyperoxic period relative to normoxia. To further examine whether this effect was due to a power change in the evoked response itself or reflected a change in the underlying spontaneous activity, we conducted an analysis similar to that used for the EEG data during the pre-task period (500ms before the onset of the visual stimulus). Interestingly, during the pre-task period, α and β signal power also decreased (by 13.6% ±3.5%, P = 0.03, and 11.6% ± 3.6%, P = 0.03, respectively), suggesting that hyperoxia caused a reduction in α and β power regardless of the presence of task simulation. The δ, θ, and γ bands did not show any significant difference in either the pre-task or the task periods.

Response time did not show a significance difference between normoxic and hyperoxic states.

## Discussion

This study investigated the influence of hyperoxia on neural electrophysiological activity in the brain under resting-state and during the performance of a visual task. Our data suggested that hyperoxia, via inhalation of O_2_-enriched gas, has a pronounced effect on neural activity. Specifically, hyperoxia decreased the α and β band power of spontaneous neural activity. For task-evoked neural activity, hyperoxia resulted in a slower appearance of ERP signal peaks, when compared to normoxia.

Oxygen therapy is widely used for several clinical conditions. However, most of the conditions in which oxygen treatment has demonstrated a definitive benefit are related to wound-healing in diabetes and cancer patients [2], but were not associated with brain function. Indeed, the proposed use of oxygen in brain diseases, such as autism, cerebral ischemia, brain injury, and cerebral palsy, is still controversial [3–7,9]. Similarly, the effect of oxygen inhalation on cognitive performance is also a matter of debate [24–27]. Consequently, a neurobiological understanding of the impact (positive or negative) of O_2_ on the brain is urgently needed.

Due to the intimate relationship between oxygen and hemodynamic signals, conventional functional imaging techniques, such as BOLD fMRI, are contaminated by the direct effect of oxygen, and thus, are not suitable for the examination of neural activity changes under hyperoxia. Therefore, the number of human studies of the effects of hyperoxia on neural activity is limited. Several early studies have reported that hyperoxia does not alter resting-state EEG or evoked potentials [15,28]. With advances in neuroimaging technologies, recent evidence has suggested a potential effect of hyperoxia on neural activity. Xu et al. [13] utilized MRI-based methods to measure metabolic responses to hyperoxia, and found that hyperoxia with 98% FiO2 decreased the cerebral metabolic rate of oxygen (CMRO_2_) by 10.3±1.5%. Richards et al. [29] measured oxygen metabolism using a ^13^C NMR technique and revealed that oxygen treatment in a canine model of ischemia reduced O_2_ metabolism. Using MEG, Croal et al. found that α, β, and lower γ bands in the occipital lobe showed a power reduction of 3–5% [16]. The observations of the present study are in general agreement with these prior reports and showed that EEG rhythms associated with cognitive processing, such as the α and β bands, showed a diminishment under hyperoxic conditions. Our findings involve a larger portion of the brain compared to that studied by Croal et al., which was more localized to the occipital lobe [16]. This discrepancy could be due to the poorer spatial localization of the EEG technique, relative to MEG. The changes observed in the present study appear to be reproducible and were noted in three separate experimental settings: 1) the resting state; 2) the interval between task performance; and 3) during task performance. Furthermore, the present study also examined evoked-related potentials and revealed that the response was delayed during hyperoxia. Collectively, these data provide further support for a neural modulation effect of hyperoxia on the brain.

Our data suggest that the visual ERP is delayed by 1–2 ms. This difference may be too small to detect in the response time, which is influenced by other factors, such as muscle response time. Thus, objective markers, such as EEG, may be more sensitive than behavioral measures, e.g., response time, in terms of indexing the negative impact of hyperoxia on the brain.

The cellular and molecular underpinnings for the observed EEG changes need to be studied in future mechanistic investigations. However, one possible mechanism is oxygen toxicity. It is known that up to 2% of the oxygen consumed by our body is only partially reduced, which produces reactive oxygen species (ROS) [30]. Excessive amounts of ROS in the body can cause oxidative stress [31,32]. A high O_2_ content, i.e., hyperoxia, presumably could increase the production of ROS [33,34]. In brain slices and animal models, hyperoxia has been shown to reduce neurotransmission [35] and suppress neural sensitivity to sensory stimulus [36]. However, most prior studies showing oxidative stress have used a longer period, e.g., hours to days, of hyperoxia. Thus, it is unclear whether short-term hyperoxia on the order of minutes is sufficient to cause a detectable oxidative stress effect. If not, then other mechanisms would need to be identified to explain the findings of this and other previous studies [13,16].

The findings regarding altered neural activity also have implications in hyperoxia-calibrated fMRI. BOLD calibration using gas challenges, such as hyperoxia, has been used to estimate task-evoked neurometabolic responses [37,38] and, more recently, to measure baseline oxygen extraction fraction and cerebral metabolic rate of oxygen [10–12]. If the resting-state EEG changes observed in the present study are also accompanied by a metabolic suppression [13], this effect should be incorporated in the calibrated fMRI model,s or, more interestingly, used as an additional variable in the parameter estimation [39]. If unaccounted for, the M factor and CMRO2 in hyperoxia-calibrated fMRI could both be overestimated.

The knowledge of how hyperoxia alters neural activity is also of significance in another area of public health. Millions of divers, as well as cancer and diabetes patients, receive oxygen therapy for wound-healing [1,2]. It is, therefore, important to understand and characterize the neurobiological effects of this hyperoxia-induced oxidative stress on brain function, even in the absence of clinical symptoms (e.g., seizure). Tonic alpha power is generally thought to be positively correlated with cognitive abilities, such as retrieval of long-term memory [40]. Therefore, if further verified, a reduction in alpha power due to hyperoxia may suggest a potential detrimental effect of high concentration oxygen on memory. However, more spatially-specific techniques are needed to examine such changes in relevant brain regions, such as the thalamo-cortical networks.

This study has a few limitations. First, although EEG can provide an assessment of brain neural activity, it lacks the spatial resolution that MRI or MEG can provide. Thus, our findings in this study showed minimal spatial heterogeneity in terms of the O_2_ effect on neural activity. It is possible that there is some spatial heterogeneity, but our EEG technique could not effectively detect it. A second limitation is that our data provided limited mechanistic insight into the reasons for the observed changes. For example, a measurement of the concentration of reactive oxygen species in the brain, e.g., by obtaining a lumbar puncture to draw cerebrospinal fluid (CSF) and use CSF as a surrogate for the brain tissue, before and during hyperoxia, would have been useful to ascertain the presence of oxidative stress during the short-term hyperoxia challenge applied in this study. A third limitation is that we studied only a single hyperoxic state, but did not perform graded hyperoxia or hypoxia studies, unlike some prior studies [13,41,42].

In conclusion, this study showed that normobaric hyperoxia has a significant effect on human brain activity. Spontaneous neural activity is suppressed in the α and β bands while event-related potentials are delayed in time. These effects may be relevant for calibrated fMRI, as well as for the impact of oxygen therapy on the brain and cognition.

## Supporting information

S1 Fig Bar plots of Fig 2a after removing an outlier data point. (TIF)

S1 File Data from individual subjects. (XLSX)
