# Supplementary figures and images for: The impact of hyperoxia on brain activity: A resting-state and task-evoked electroencephalography (EEG) study
Source: PLoS One. 2017 May 2;12(5):e0176610. doi: 10.1371/journal.pone.0176610 (PMC5412995; doi:10.1371/journal.pone.0176610)

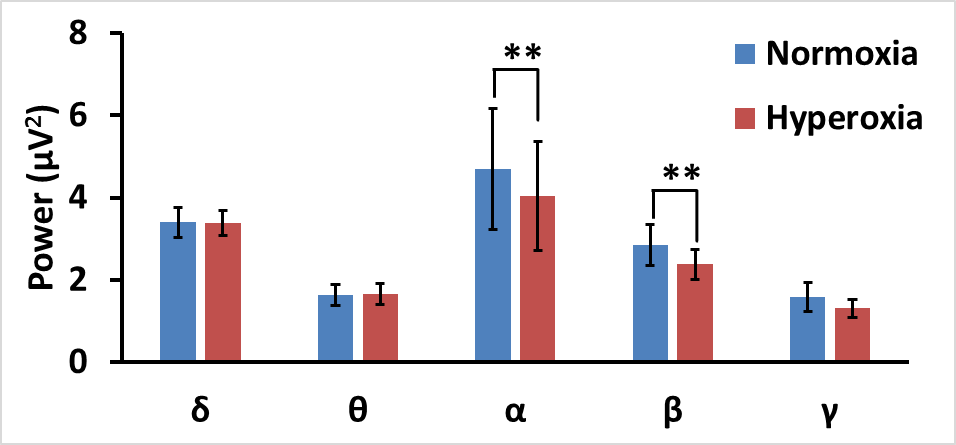

Supplement: S1 Fig — (TIF) [file pone.0176610.s001.tif]
